# Medical terminology-based computing system: a lightweight post-processing solution for out-of-vocabulary multi-word terms

**DOI:** 10.3389/fmolb.2022.928530

**Published:** 2022-08-12

**Authors:** Nadia Saeed, Hammad Naveed

**Affiliations:** Computational Biology Research Lab, Department of Computer Science, National University of Computer and Emerging Sciences (NUCES-FAST), Islamabad, Pakistan

**Keywords:** medical terminology, named entity recognition, linguistic approach, natural language processing, biomedical nomenclature, out-of-vocabulary

## Abstract

The linguistic rules of medical terminology assist in gaining acquaintance with rare/complex clinical and biomedical terms. The medical language follows a Greek and Latin-inspired nomenclature. This nomenclature aids the stakeholders in simplifying the medical terms and gaining semantic familiarity. However, natural language processing models misrepresent rare and complex biomedical words. In this study, we present MedTCS—a lightweight, post-processing module—to simplify hybridized or compound terms into regular words using medical nomenclature. MedTCS enabled the word-based embedding models to achieve 100% coverage and enabled the BiowordVec model to achieve high correlation scores (0.641 and 0.603 in UMNSRS similarity and relatedness datasets, respectively) that significantly surpass the n-gram and sub-word approaches of FastText and BERT. In the downstream task of named entity recognition (NER), MedTCS enabled the latest clinical embedding model of FastText-OA-All-300d to improve the F1-score from 0.45 to 0.80 on the BC5CDR corpus and from 0.59 to 0.81 on the NCBI-Disease corpus, respectively. Similarly, in the drug indication classification task, our model was able to increase the coverage by 9% and the F1-score by 1%. Our results indicate that incorporating a medical terminology-based module provides distinctive contextual clues to enhance vocabulary as a post-processing step on pre-trained embeddings. We demonstrate that the proposed module enables the word embedding models to generate vectors of out-of-vocabulary words effectively. We expect that our study can be a stepping stone for the use of biomedical knowledge-driven resources in NLP.

## 1 Introduction

Familiarity with medical terminology assists medical practitioners and other stakeholders like doctors, nurses, and clinicians to understand rare and complex vocabulary. The evolution of medical terminology presents challenges in promoting the use of electronic health and medical records. For example, most medical terms originate from Greek and Latin words, making reading and spelling difficult Henderson and Dorsey (2019); Banay (1948). Medical researchers acquire conceptual skills with thorough learning of medical terms, dictionaries, and references such as Merriam-Webster Merriam-Webster (2018), WebMD WebMD (2012), and MedicineNet MedicineNet (2007), etc.

The electronic health records (EHRs) contain the diagnoses, pharmacological, and drug-disease concepts that provide a complete view of a patient’s health. EHRs can inform drug discovery, treatment pathways, and real-world safety assessments. Unstructured text from EHRs can be encoded in a structured format (vectors) for downstream analysis using NLP methods. Unfortunately, the word embedding models faced the Out-of-vocabulary (OOV) words problem or used ineffective sub-word representations that caused low performance in intrinsic tasks to retrieve conceptual properties.

Popular embedding models including BERT (Devlin et al., 2019), ELMO (Peters et al., 2018), and FastText (Bojanowski et al., 2017) solve the OOV problem by using pre-processing tokenization techniques based on WordPiece (Wu et al., 2016), characters, and n-grams. These traditional NLP approaches are not built to understand the unique vocabulary and grammar of medical texts. For example, *mastodynia* is a disease whose meaning can be approximated from related and simple words like *breast, pain, and discomfort* rather than to approximate it with its non-logical sub-words or n-grams like [CLS],mast,##ody, and ##nia [SEP].

Biomedical and clinical terms have unique and complex characteristics such as prefixes, roots, suffixes, etc., therefore requiring a more focused effort around methodologies within the medical NLP domain (Banay, 1948; Meystre et al., 2008; Cohen and Demner-Fushman, 2014; Leaman et al., 2015; Henderson and Dorsey, 2019). In recent years, biomedical and clinical embedding models such as BioWordVec (Zhang et al., 2019) and BioNLP (Chiu et al., 2016) models have been trained under low capacity resource requirements like the Gensim library (Řehřek and Sojka, 2011). However, these models generally follow the Word2Vec (Mikolov et al., 2013a; Mikolov et al., 2013b) and GloVe (Pennington et al., 2014) algorithms, which face the OOV problem. The embedding models trained using the FastText algorithm (Bojanowski et al., 2017) claim to have solved the OOV problem, however they are ineffective.

The pre-trained embedding models generate either context-sensitive or distributed representations of word vectors. The context-sensitive models generate multiple embeddings for a word that capture the context based on its positional encoding learned using transformers or recurrent neural networks (RNN). Bidirectional Encoder Representations from Transformers (BERT) is a popular embedding model (Devlin et al., 2019), that has been extended to clinical and biomedical domains [ClinicalBERT Huang et al. (2019) and BioBERT Lee et al. (2020)]. These models tackle the OOV problem with the WordPiece algorithm (Wu et al., 2016) that represents a word by its frequent sub-words, e.g., immunoglobulin → (i,mm,uno,g,lo,bul,in). Embeddings from Language Models (ELMO) is another context-sensitive model that generates word-level embeddings using multiple convolutional neural networks (CNNs) with bi-directional LSTM (BiLSTM) (Peters et al., 2018). ELMO has also been extended to generate biomedical and clinical embeddings (Zhu et al., 2018; Jin et al., 2019; Subramanyam and Sangeetha. 2020). These studies deal with the OOV problem through character-level embeddings. Boukkouri et al. showed that character-level embedding was a better approach to removing biases in sub-words for biomedical terms than WordPiece e.g., choledocholithiasis → (cho,led,och,oli,thi,asi,s) (Boukkouri et al., 2020; Wu et al., 2016). The context-sensitive models are expensive, both in terms of computational and space resources since they train millions of hyperparameters with multiple attention heads.

The distributed representation models learn embeddings based on the word usage in a given corpus. The resultant vectors capture the contextual similarity between words. These static models generate a single vector per word and are trained either under Word2Vec (Mikolov et al., 2013b), GloVe (Pennington et al., 2014), or FastText (Bojanowski et al. (2017)). FastText enriches each word vector with its respective n-grams. It handles the OOV problem by leveraging the sum of n-gram vectors of the unknown word, e.g., n = 3, myocarditis →< my, myo, yoc, oca, car, ard, rdi, dit, iti, tis, is > . On the other hand, the embedding models trained by Word2Vec and GloVe face the OOV problem. These models replace unknown words with tags such as < UNK > or a randomly generated vector, where different unknown words lose their uniqueness.

In this study, we proposed MedTCS, a novel medical terminology-based module that assists the pre-trained embedding models to generate vectors for unknown words and compound terms. It is an innovative post-processing solution that explores the given search space for those terms that are not directly present but whose semantic information is. MedTCS turns the word into its meaningful sub-words using the biomedical segmentation model. Ultimately, MedTCS helps the distributed representation models handle the OOV problem effectively.

We have compared MedTCS with recent state-of-the-art embedding models to investigate the effectiveness of capturing semantic information without encountering OOV problems. Our results showed that MedTCS enhanced the performance of pre-trained models significantly in terms of coverage and/or semantic correlation. Moreover, we conducted experiments to assess the usefulness of enriched embedded vectors for downstream NER tasks (disease name identification and drug indication classification). MedTCS performed better than FastText in terms of performance and resource consumption on all tasks. The MedTCS module enhanced the performance of the FastText word vectors as compared to the n-gram and sub-word approaches used for unknown words (Flamholz et al., 2022). Furthermore, MedTCS is extensible with new terminologies and content.

## 2 Methodology

MedTCS is a lightweight module implemented in Python. It is a knowledge-driven system for forming terms by pluralizing, singularizing, and deconstructing words.

### 2.1 Meta-data collection

#### 2.1.1 Word component dictionary

**FIGURE 1:**
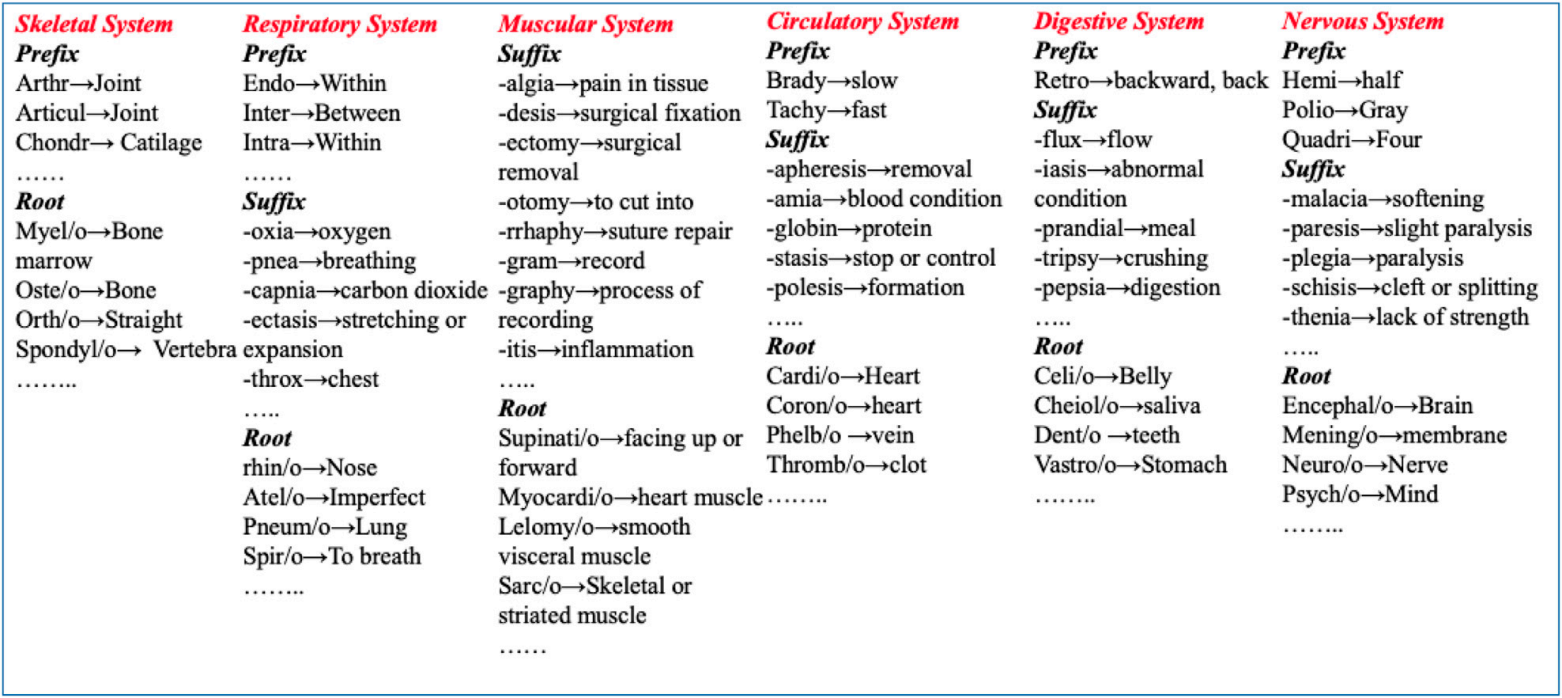
Understanding biomedical terms by mapping term components to human organ system.

In MedTCS, we build meta-dictionaries for the prefixes, roots, and suffixes defining the meanings of medical term components. In addition to the lexical normalization and plural conversion of the unknown term, we have developed medical terminology-based look-up dictionaries for the parser by collecting information from “Medical Terminology for Dummies” (Henderson and Dorsey, 2019). The three semantic dictionaries contain 467 root words, 432 prefixes, and 112 suffixes, along with their corresponding meanings as shown in Figure 1.

#### 2.1.2 Word segmenter model

MedTCS used Morfessor as a word segmenter model (Smit et al., 2014). In order to train the semi-supervised Morfessor 2.0 model, we used a corpora of 240 k words consisting of medical academic word list, e-biology, e-chemistry, and NLTK words (Bird and Loper, 2004; Wang et al., 2008).

### 2.2 MedTCS framework

**FIGURE 2:**
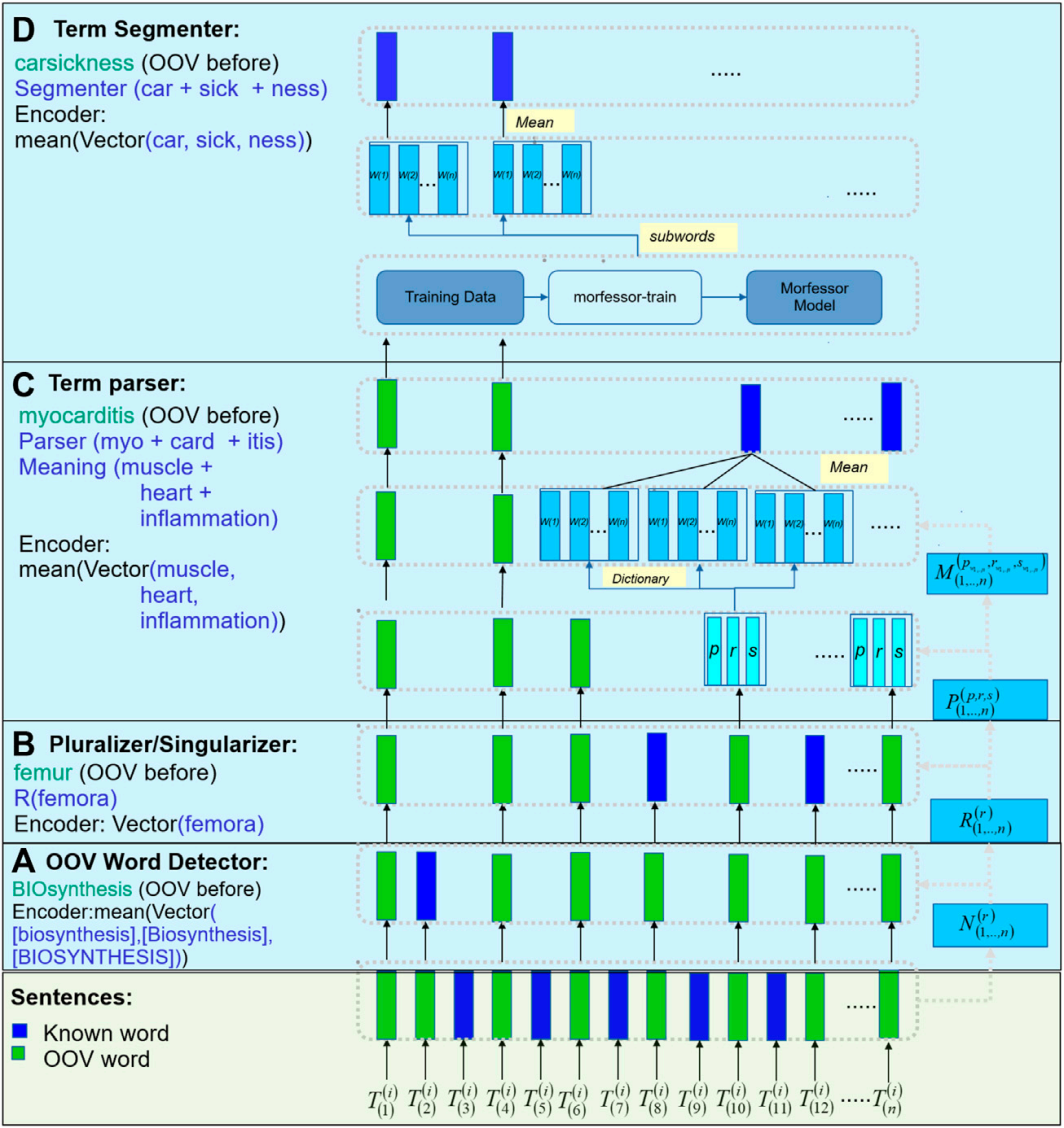
MedTCS framework: **(A)** MedTCS detector normalizes the unknown terms and search in vocabulary; **(B)** Rule-based pluralizer or singularizer sub-module used to normalize the unknown terms; **(C)** Architecture for term-parser, where the compound words encode for its components that infer from the dictionary for its semantic words that encode as its mean vector; **(D)** Architecture for term segmenter, a pre-trained segmentation model segments the word into subwords that encodes as its mean vector.

Figure 2 provides a high-level description of our MedTCS module to encode OOV words from a set of sentences or words. In step (a), the OOV words are normalised for multiple morphological rules (represented as $N_{1,\ldots ,n}^{r}$ ). In step (b), the remaining OOV words are exchanged with its plural or singular form by applying medical terminology-based rules (represented as $R_{1,\ldots ,n}^{r}$ ). At each step, the normalized terms are encoded into vectors. In the succeeding steps (c) and (d), the words are passed to the parser, where dictionaries of prefix *p*, root *r,* and suffix *s* are used to tokenize them (represented as $P_{1,\ldots ,n}^{p,r,s}$ ). Each component of the term is replaced with their respective meaning in the dictionary as a word list (represented as $M_{1,\ldots ,n}^{p_{w_{i},..w_{k}},r_{w_{i},..w_{k}},s_{w_{i},..w_{k}}}$ ). The encoder encodes the tokens into its mean vector. Finally, the remaining non-encoded words are passed to the pre-trained term segmenter model to intra-tokenize into meaningful words (that are also encoded as mean vectors).

#### 2.2.1 MedTCS OOV word detector

The MedTCS OOV word detector identifies whether a token is known or unknown for a given vocabulary. The unrecognized word is passed through multiple normalization steps: 1) lexical property of the alphabetic case is applied, 2) intra-term punctuation marks are retained while ignoring starting and ending symbols, and 3) apostrophe symbols for OOV word detection are normalized.

#### 2.2.2 MedTCS pluralizer/singularizer

The MedTCS pluralizer is based on the plural rules defined in medical terminology and implemented as a finite state machine. The sigularizer acts as a reverse finite state machine of the pluralizer.

#### 2.2.3 MedTCS term parser

The MedTCS term parser was applied to an unknown word in two parts. First, the rule-based parser breaks the word into components of medical terminology, i.e., root, prefix, and suffix. Second, this parser implemented a dictionary lookup algorithm on each component to map its meaning. These dictionaries contained the definitions of the components of the medical terms collected from medical notes (Banay, 1948; Cohen and Demner-Fushman, 2014; Henderson and Dorsey, 2019). Each component in the dictionary belonged to one of the following human organ systems as shown in Figure 1 e.g., -pnea → breathing was a suffix belonging to the respiratory system. The root component is normalized for its combined form, like pneum/o → lung. Each component incrementally contributes in generating the vector representation of the unknown word. Each discovered vector by MedTCS term parser belonged to the lexical part of the unknown word and had attributes defined in the medical terminology. For example, *choledocholithiasis* → [“choledoch” (prefix)]+[“o”]+[“lithiasis” (suffix)] → [common bile duct]+[calculus or stone]. In case the term parser does not return a valid vector, the term segmenter was executed to determine meaningful sub-words of the unknown word.

#### 2.2.4 MedTCS term segmenter

The MedTCS term segmenter is a wrapper around the Morfessor 2.0 module to acquire the meaningful sub-word units of an unknown term (Virpioja et al., 2013; Smit et al., 2014). We trained the system on a subset of Biology, Chemistry, and English corpora. Our word-level segmentation system returned the average vector of meaningful sub-words of an unknown term (like *seasickness* → sea + sick + ness).

### 2.3 Datasets

In addition to the widely tested UMNSRS similarity and relatedness datasets (Pakhomov et al., 2010), and the MyoSRS dataset (Pakhomov et al., 2011), our intrinsic evaluation included the latest and comparatively large benchmark named the EHR-RelB dataset Schulz et al. (2020). These datasets consist of word pairs with their similarity or relatedness scores assigned by medical experts.

We checked the applicability of the MedTCS module to extract disease names from two publicly available datasets [NCBI-Disease and BC5CDR-Disease, Wang et al. (2019)] using the *BIO* scheme. *BIO* is used to encode entity annotations as token tags, where *B* indicates the beginning of the phrase, *I* is the element within the phrase, and *O* is the element outside of the phrase. Table 1 gives the details of benchmark datasets used for performance evaluation. We also used the Drug Indication Classification and Encyclopedia (DICE) dataset Bhatt et al. (2021) to check the performance enhancement achieved by MedTCS on classifying a sentence into indication or non-indication defined for five categories (indications, contradictions, side effects, usage instructions, and clinical observations). The dataset contained 7,231 sentences that were categorized into 4,297 indications, 1,673 clinical observations, 701 contraindications, 492 usage instructions, and 68 side effects.

**TABLE 1:** Statistics of Datasets*.*

Evaluation	Dataset	Corpus size	Type
Intrinsic Evaluation	UMNSRS-similarity Pakhomov et al. (2010)	566 term pairs	Pairwise similarity
	UMNSRS-relatedness Pakhomov et al. (2010)	588 term pairs	Pairwise relatedness
	MyoSRS Pakhomov et al. (2011)	101 term pairs	Pairwise relatedness
	EHR-RelB Schulz et al. (2020)	3630 term pairs	Pairwise relatedness
Extrinsic Evaluation	Dataset		
	BC5CDR Wang et al. (2019)	1500 articles	Disease Name
	NCBI-Disease Wang et al. (2019)	793 abstracts	Disease Name
	DICE Bhatt et al. (2021)	7231 sentences	Drug Indication

All the datasets discussed in Table 1 are publicly available in split form.

### 2.4 Evaluation metrics

In NLP, intrinsic evaluation extracts the semantic properties of pre-determined ground truth concepts with encoded vectors. On the other hand, extrinsic evaluation decodes the encoded information of embedding models and evaluates their efficiency in performing downstream tasks like NER. For the extrinsic evaluation, the coverage percentage is based on the number of encoded tokens of a dataset with the respective embedding model.

$$cosine\text{\_}similarity\left(A,B\right)\;=\;\frac{A.B}{\left\|A\|\times \|B\right\|},$$ (1)

$$recall\;=\;\frac{TruePositives}{TruePositives\;+\;FalseNegatives},$$ (2)

$$precision\;=\;\frac{TruePositives}{TruePositives\;+\;FalsePositives},$$ (3)

$$F1\text{\_}score\;=\;\frac{2\times \left(precision\times recall\right)}{precision\;+\;recall}.$$ (4)

In intrinsic evaluation, the similarity scores are computed between the encoded term pairs using the cosine similarity as given in Eq. 1. Furthermore, these similarity scores are used with the rankings by human experts to compute the Spearman (Sp) correlation coefficients with SciPy (Virtanen et al., 2020).

In extrinsic evaluation, the task of tagging the biomedical entities is performed by using a machine learning model trained on the encoded vectors. The performance measures used for this are recall, precision, and F1_score (Eqs 2–4).

## 3 Results

We compared the semantic and conceptual functionality of MedTCS with the pre-trained sub-word models (derivative models of BERT Devlin et al. (2019) and FastText Bojanowski et al. (2017)) for the biomedical and clinical domains.

### 3.1 Intrinsic evaluation

We evaluated the capability of MedTCS to enable the pre-trained word embedding models for encoding the OOV terms. MedTCS assisted the pre-trained word embedding models to achieve full coverage of all the conceptual pairs in the datasets. Moreover, we compared our model with related embedding models trained with FastText and BERT algorithms.

In this experiment, we included popular embedding models as a baseline, such as BioWordVec (Zhang et al., 2019), BioNLP (Chiu et al., 2016), PubMed-w2v, PubMed-PMC-w2v, and Wiki-PubMed-PMC-w2v (Moen and Ananiadou, 2013), most of which are defined under the Word2Vec algorithm (Mikolov et al., 2013b). Our baseline has the same encoder and decoder method for the NLP task without the MedTCS module. As the selected datasets include multi-word terms; therefore, the average vectors of each word are calculated with and without the MedTCS module.

**FIGURE 3:**
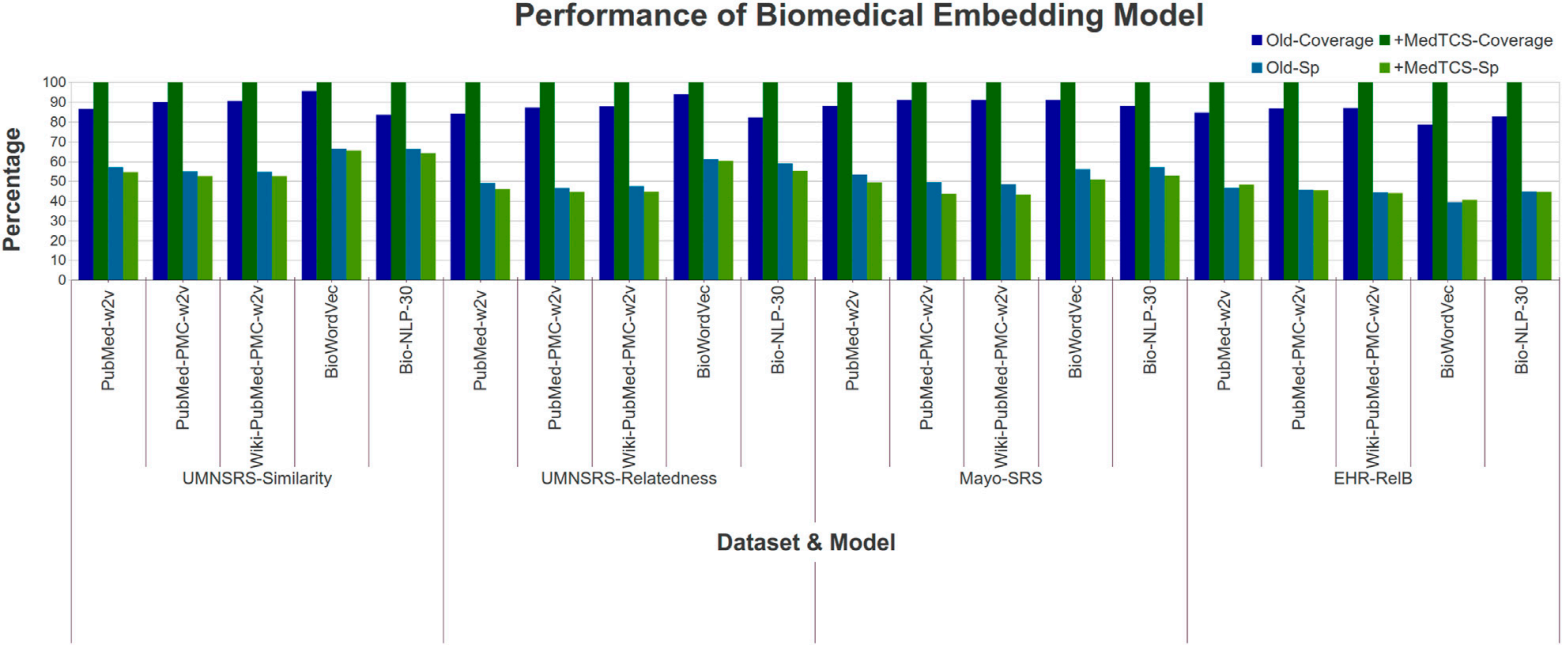
Comparison of performance variations in biomedical embedding model after adding MedTCS module on datasets of Table 1 for intrinsic evaluation.

Our analysis showed that the MedTCS module enabled all pre-trained embedding models to achieve full coverage with persuasive correlation scores on all datasets (Figure 3). For example, on the EHR-RelB dataset, the coverage of the *BiowordVec model* was enhanced from 2,857 terms pair to 3,630 terms pair and the Spearman (Sp) correlation also improved from 0.393 to 0.405. Overall, our results show that all models achieved 100% coverage of all the datasets with a slight decrease in correlation scores. As the OOV words are being approximated, therefore, a slight decrease in correlation scores is naturally expected.

We also enhanced the latest clinical word embedding models with MedTCS. The PMC Open Access Subset-Case reports (OA-CR) embedding models trained using word2vec/GloVe encountered the OOV word problem while working on the UMNSRS-Similarity dataset (Flamholz et al., 2022). MedTCS improved the coverage of all word embedding models from approximately 62% → 98% Supplementary Case S1.

Similarly, we analyzed the functionality of FastText to handle the OOV problem on the PMC Open Access subsets - Clinical Report (OA-CR) models and the PMC Open Access subsets - all manuscripts (OA-All) models (Flamholz et al., 2022). FastText trains each word vector along with its n-gram vectors. In the case of any OOV word, the average of its n-gram vectors are used to encode it (Bojanowski et al., 2017). For the FastText based OA-CR-600 embedding model, the Spearman (Sp) correlation value improved from 0.38 → 0.47 Supplementary Case S2. In conclusion, the MedTCS module enabled the different variants of OA-CR models to encode the vector for OOV terms from its search space effectively.

**FIGURE 4:**
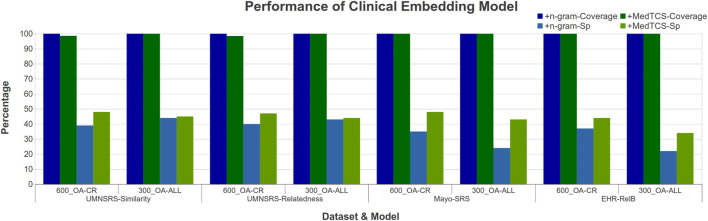
Comparison of performance variations in clinical embedding model after adding MedTCS module on datasets of Table 1 for intrinsic evaluation.

The OA-CR models have a small vocabulary; MedTCS enabled these models to achieve 100% coverage on all datasets as shown in Figure 4. Moreover, the MedTCS assisted the OA-CR models and the OA-ALL models to have significantly improved correlation scores, e. g., the FastText OA-All-300d model on the EHR-RelB dataset achieved 100% coverage and improved the Spearman (Sp) correlation scores from 0.25 → 0.35. The results on other variants of the OA-CR and OA-ALL embedding models for intrinsic evaluation are similar, as shown in Supplementary Figure S3.

On the other hand, BERT models use sub-words to solve the OOV word problem. We compared the proposed model with BERT and its derivative models defined for the clinical and biomedical domain [available on HuggingFace Wolf et al. (2019); Wolf et al. (2020)]. MedTCS outperformed BERT-based models by a significant margin in terms of correlation scores on the UMNSRS-Similarity dataset (Table 2). Moreover, in (Table 3), we have compared our best achieved results with recently reported scores of UMNSRS datasets (Mao and Fung, 2020; Singh and Jin, 2020; Yuan et al., 2022). MedTCS achieved significantly better coverage and correlation scores.

**TABLE 2:** Comparison of sub-word embeddings with word embedding + MedTCS on the UMNSRS-Similarity datasets.

Model		Version	Sp
BERT	BERT Devlin et al. (2019)	bert-base-uncased	0.07
	BioBert Lee et al. (2020)	dmis-lab/biobert-v1.1	0.30
	BlueBert Peng et al. (2019)	bionlp/bluebert_pubmed_mimic_uncased_L-12_H-768_A-12	0.36
	Bio_ClinicalBERT	emilyalsentzer/Bio_ClinicalBERT	0.23
Model	Alsentzer et al. (2019)	allenai/scibert_scivocab_uncased	0.18
	SciBERT Beltagy et al. (2019)		
	PubMedBERT Gu et al.(2022)	microsoft/BiomedNLP-PubMed BERT-base-uncased-abstract-fulltext	0.23
	CODER Yuan et al. (2022)	GanjinZero/UMLSBert_ENG	0.47
Word2Vec	PubMed-w2v	PubMed-w2v.bin	0.52
	+MedTCS		
	PubMed-PMC-w2v	//	0.49
Model	+MedTCS		
	Wiki-PubMed-PMC-w2v	//	0.49
+	+MedTCS		
	Bio-NLP-30 Chiu et al. (2016)	Bio-NLP-30	0.63
MedTCS			
	+MedTCS		
	BioWordVec Zhang et al. (2019)	BioWordVec	0.64
	+MedTCS		

**TABLE 3:** Comparison of the word embedding + MedTCS best scores with latest reported results.

Model	UMNSRS-Similarity		UMNSRS-Relatedness		Model description
	
	#566	Sp	#587	Sp	
BioWordVec+	480	0.629	473	0.590	A combined model of Graph
Graph					convolutional network (GCN)
Embeddings					a path-based graph embedding
(GCN) Mao and Fung (2020)					with BioWordVec embedding
Context2Vec+	471	0.634	484	0.561	Composite model of contextual
BioWordVec+					embedding with BioWordVec
PubMed + PMC					concatenated with PubMed and
Singh and Jin (2020)					PMC word embedding to
					achieve these results
CoderBERT	543	0.543	564	0.473	A BERT-based model obtained
Kalyan and Sangeetha (2021)					by fine-tuned a pre-trained
					BioBERT on UMLS
					synonyms and relations
SapBERT-S	543	0.585	564	0.505	A BERT-based model fine-tuned
Kalyan and Sangeetha (2021)					a pre-trained PubMedBERT on
					UMLS using a self-alignment
					objective to cluster the term
					concept
BioWordVec	**566**	**0.641**	**587**	**0.603**	BioWordVec with our composed
+MedTCS					MedTCS module, to extract the
					vector representation of a known
					and unknown term

Results with highest values of correlation and coverage scores are shown in bold.

### 3.2 Extrinsic evaluation

Extrinsic evaluation requires training a system for the related downstream NLP tasks like NER, classification, etc,. The existing word embedding models achieve sub-optimal results due to the ineffective handling of OOV words (encoded unknown words with their n-gram vectors or a randomly generated vector). We tested the enriched vectors (by MedTCS) in identifying disease names from documents. We trained a bidirectional long-short term memory with a convolutional neural network (BiLSTM-CNN) Chiu and Nichols (2016) on the annotated corpus of BC5CDR and NCBI-disease (Table 1).

**FIGURE 5:**
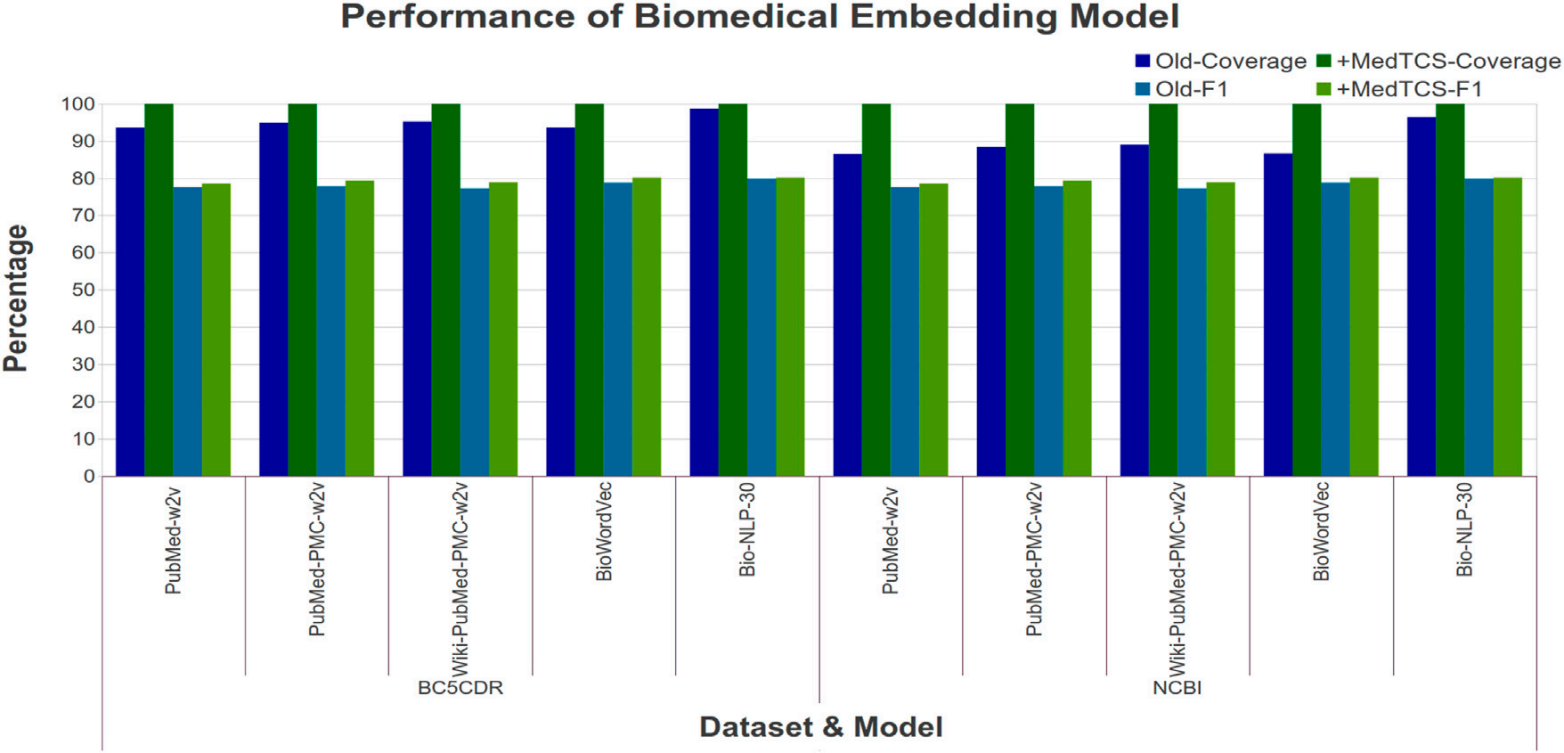
Comparison of performance variations in biomedical word embedding model after adding MedTCS module on datasets of Table 1 for NER task.

Figure 5 showed the performance enhancement in terms of coverage and F1 score (in percentage) achieved after replacing the randomly generated vector approach with our MedTCS module for OOV words. The MedTCS module with PubMed-w2v embedding enabled improved the coverage up to 13% on the NCBI-Disease dataset. Overall, MedTCS enabled word embedding models to achieve 100% coverage with an improved F1 score.

**FIGURE 6:**
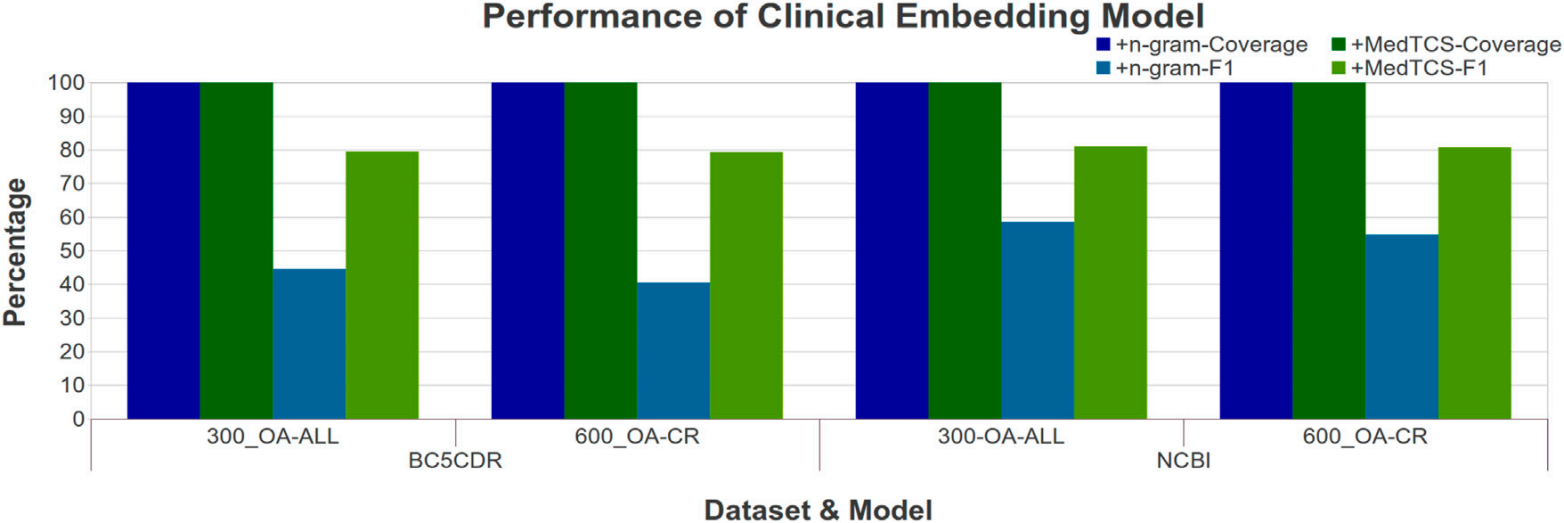
Comparison of performance variations in clinical FastText embedding model after adding MedTCS module on datasets of Table 1 for NER task.

Similarly, we compared the MedTCS module with the n-gram approach for the NER task. Figure 6 showed that MedTCS improved the F1-score between 10 and 20% for the various embedding models as compared to the FastText n-gram vectors under the same parameters as for the BiLSTM NER system (Chiu and Nichols, 2016). The FastText OA-All-300d model with MedTCS achieved an F1-score of 0.80 (an improvement of 0.35) on the BC5CDR corpus and an F1-score of 0.81 (an improvement of 0.32) for the NCBI-disease corpus. Similar results were achieved on the other variants of the OA-CR and OA-ALL embedding models for the NER task (Supplementary Figure S4).

**FIGURE 7:**
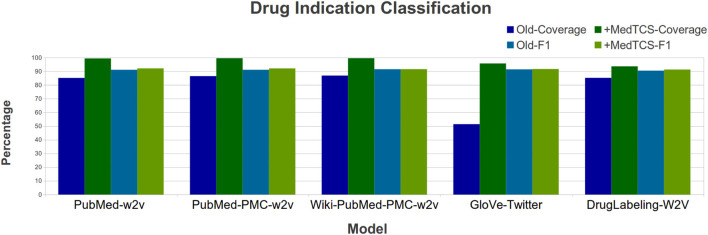
Model performances enhanced with MedTCS for Drug indication classification.

Bhatt et al. (2021) recently developed a Drug Indication Classification and Encyclopedia (DICE) based on FDA approved human prescription drug labeling. They also generated “DrugLabelling-W2V” embeddings based on Word2Vec and used them to classify each sentence into one of the five classes (indications, contradictions, side effects, usage instructions, and clinical observations). We enhanced the “DrugLabelling-W2V” embedding with the MedTCS module and improved the coverage by 9% and the F1_score by 1% (Figure 7).

## 4 Discussion

Curating a large corpus is the traditional approach in NLP to cover more concepts and enhance the vocabulary of word-level embedding models. For example, meta-data from dictionaries, meta-thesaurus, and hierarchical relationships from ontologies were also used as corpus. In the biomedical and clinical domains, the larger corpus of PubMed-PMC from MEDLINE and Wikipedia (Denoyer and Gallinari, 2006) was used to enlarge the vocabulary. Similarly, the NCBI sources, including the Medical Subject Heading (MeSH) (Lipscomb, 2000), the Unified Medical Language System (UMLS) metathesaurus concepts (NLM, 2004), and the Systemized Nomenclature of Medicine—Clinical Terms (SNOMED CT) concepts (Donnelly, 2006) have also been used as meta-corpus. The semantic content of the ontologies and the meta-data like Web Ontology Language (OWL) has also been used to train embedding vectors (Grau et al., 2008). In spite of these efforts, while encoding some rare terms and concepts, the embedding models still faced the OOV problem like in the BioWordVec embedding model (Zhang et al., 2019).

We have developed MedTCS, a module that generates the vector representation for unknown words based on medical knowledge. Different approximation techniques derived from medical knowledge bases have been used to encode the OOV words. To the best of our knowledge, this is the first-ever post-processing and run-time solution for the OOV problem that is specifically designed for pre-trained biomedical/clinical word embedding models. Each OOV word is parsed into its components, which are replaced with their meanings to generate the semantic vectors. In addition, MedTCS’s segmentation model tokenizes compound words into its word units, as shown in Figure 2. The MedTCS module outperforms the FastText n-gram approach to handle OOV words as shown in Figure 4.

In an empirical analysis of the BERT and its derivative models, we have observed that these models can have a high cosine similarity value between pairs given in datasets (Table 1). However, in the task to measure the degree of contextual relatedness and similarity between biomedical and clinical terms, they showed decreased performance (Table 2). Furthermore, according to our findings on BERT models, CODER Yuan et al. (2022) has better performance, probably because it encodes most of the words without splitting them into their sub-words, as shown in Table 4.

**TABLE 4:** Examples of the sub-word tokenization schemes followed by the different algorithms with the medical terminology-based MedTCS module.

Term	MedTCS	FastText	BioBert	CODER
		Bojanowski et al. (2017)	Lee et al. (2020)	Yuan et al. (2022)
mastodynia	breast, pain	<ma,mas,ast	[CLS],mast,##	[CLS],mast,##
	discomfort	sto,tod,ody	ody,##nia, [SEP]	odynia, [SEP]
		dyn.yni,nia,ia>		
prostatism	prostate, gland	<pr,pro,ros,ost,sta	[CLS],pro,##sta	[CLS],prost,##
	state,of,or,condition	tat,ati,tis,ism,sm>	##tism, [SEP]	atism, [SEP]
prostatorrhea	prostate, gland	<pr,pro,ros,ost.sta	[CLS],pro,##sta	[CLS],prost,##
	flow, excessive	tat,ato,tor,orr,rrh	##tor,##r,##hea	ator,##rh,##ea
	discharge	rhe,hea,ea>	[SEP]	[SEP]
blepharospasm	eyelid,or,eyelash	<bl,ble,lep,eph,pha	[CLS],b,##le,##	[CLS],ble,##
	sudden,or	har,aro,ros,osp,spa	pha,##ros,##	pha,#rosp,##
	involuntary	pas,asm,asm>	pas,##m, [SEP]	asm, [SEP]
dyslipidemia	painful,fat,a	<dy,dys,ysl,sli,lip	[CLS],d,##ys,##	[CLS]
	blood, condition	pii,pid,ide,dem	lip,##ide,##	dyslipidemia
		emi,mia,ia>	mia, [SEP]	[SEP]
dyspnea	painful, breathing	<dy,dys,ysp,spn	[CLS],d,##ys,##	[CLS],dyspnea
		pne,nea,ea>	p,##nea, [SEP]	[SEP]
urethrorrhea	urethra, flow	<ur,ure,ret,eth,thr	[CLS],u,##ret,##	[CLS],ureth,##
	excessive	hro,ror,orr,rrh,rhe	hr,##or,##r,##	ro,##r,##rh,##
	discharge	hea,ea>	hea, [SEP]	ea, [SEP]
arteriosclerosis	artery, hardening	<ar,art,rte,ter,eri	[CLS],art,##eri	[CLS],arterio
		rio,ios,osc,scl,cle	##os,##cle,##	##sc,##ler,##
		ler,ero,ros,osi,sis,is>	rosis, [SEP]	osis, [SEP]
dermatitis	Skin	<de,der,erm,rma	[CLS],der,##mat	[CLS]
	inflammation	mat,ati,tit,its,ts>	##itis, [SEP]	dermatitis
				[SEP]

Word embedding models are of great importance for various biomedical NLP applications, however they currently face a major problem of assigning vectors for unknown and rare words. To fill this gap, we have developed the MedTCS module to facilitate the pre-trained word representation models in encoding medical terms. We hope that our module will be considered as a standard medical term tokenizer for the application of NLP in the biomedical domain. MedTCS can also allow other biomedical NLP researchers to develop knowledge-based modules in a variety of real-world applications. Moreover, our research highlighted that there is a need to not only train large embedding models but also some knowledge-driven modules for the medical and clinical domains. According to our knowledge, MedTCS is the first post-processing and run-time solution for the OOV problem that improves the applicability and semantic efficiency of pre-trained embedding of medical terms.

## Data Availability

The code for our module and instructions for the use can be found on GitHub, https://github.com/NadiaSaeed/MedTCS.git. Publicly available datasets used for performance evaluation and analysis during the current study are available at https://github.com/cambridgeltl/MTL-Bioinformatics-2016. The original contributions presented in the study are publicly available. The generated dictionaries of MedTCS are available on GitHub, https://github.com/NadiaSaeed/MedTCS.git.
