# Supplementary material for: Medical terminology-based computing system: a lightweight post-processing solution for out-of-vocabulary multi-word terms
Source: Front Mol Biosci. 2022 Aug 12;9:928530. doi: 10.3389/fmolb.2022.928530 (PMC9411640; doi:10.3389/fmolb.2022.928530)
Supplement: Supplementary file 1 [file DataSheet1.PDF]

Supplementary Material

Correspondence*:
Hammad Naveed *
hammad.naveed@nu.edu.pk

1 CASE STUDY:USING MEDTCS FOR CLINICAL EMBEDDING MODELS

In natural language processing (NLP), the semantic similarity of term pairs evaluates by using their encoded vectors. While encoding, the word embedding model faced a major problem of out-of-vocabulary(OOV) terms. Flamholz et al. (2022) encountered OOV terms in UMNSRS-Similarity dataset (Pakhomov et al. (2010)). The authors reported the coverage and OOV terms in their supplementary material link. To demonstrate the advantage of a medical terminology-based OOV solution, MedTCS enhanced the clinical embedding models Flamholz et al. (2022).

1.1 Case 1: Enhanced the Coverage of Word Embedding Models

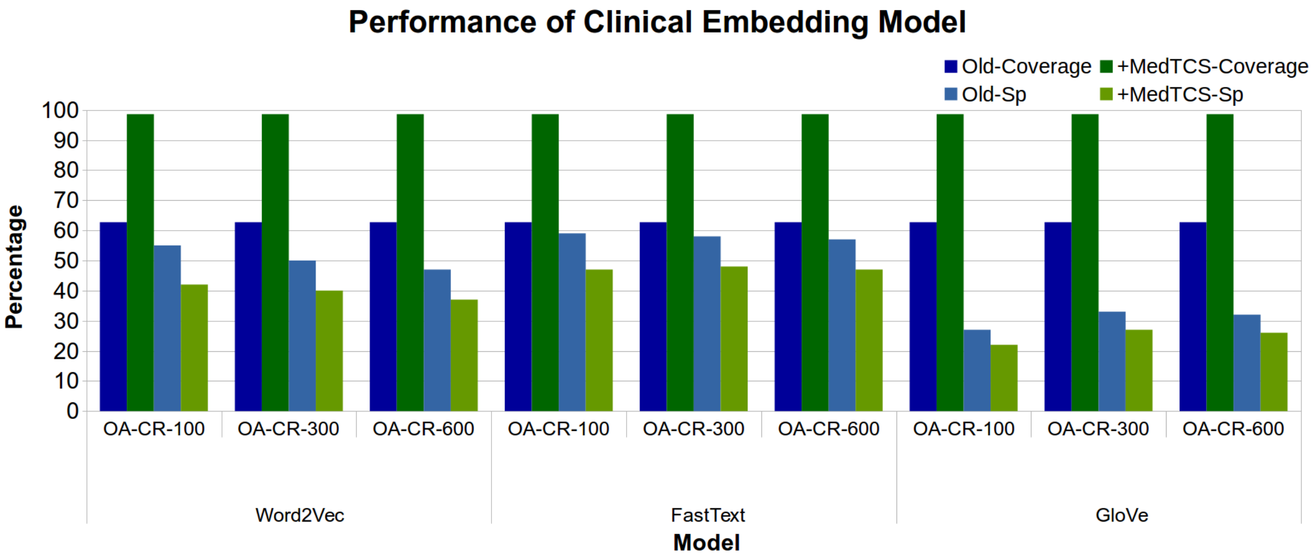

Figure 1. *Comparison of performance variations in clinical embedding model after adding MedTCS module on UMNSRS-Similarity dataset.*

The *PMC Open Access Subset- Case reports (OA-CR)* corpus based embedding was trained under Word2Vec and GloVe algorithm with 100, 300, and 600 embedding dimensions and a vocabulary of 333,360 tokens. These models failed to compute the similarity scores of 211 terms pairs out of 566 total pairs of UMNSRS-Similarity dataset Pakhomov et al. (2010), while MedTCS precisely encoded these term pairs and improved coverage from 62% $\rightarrow$ 98% as in Figure 1.

1.2 Case 2: Compared MedTCS with the FastText(n-gram) Approach

The FastText OA-CR model trained with n-grams vectors resolved the OOV problem. However in practice, it provided an ineffective vector for unknown words. Whereas MedTCS estimated vectors for

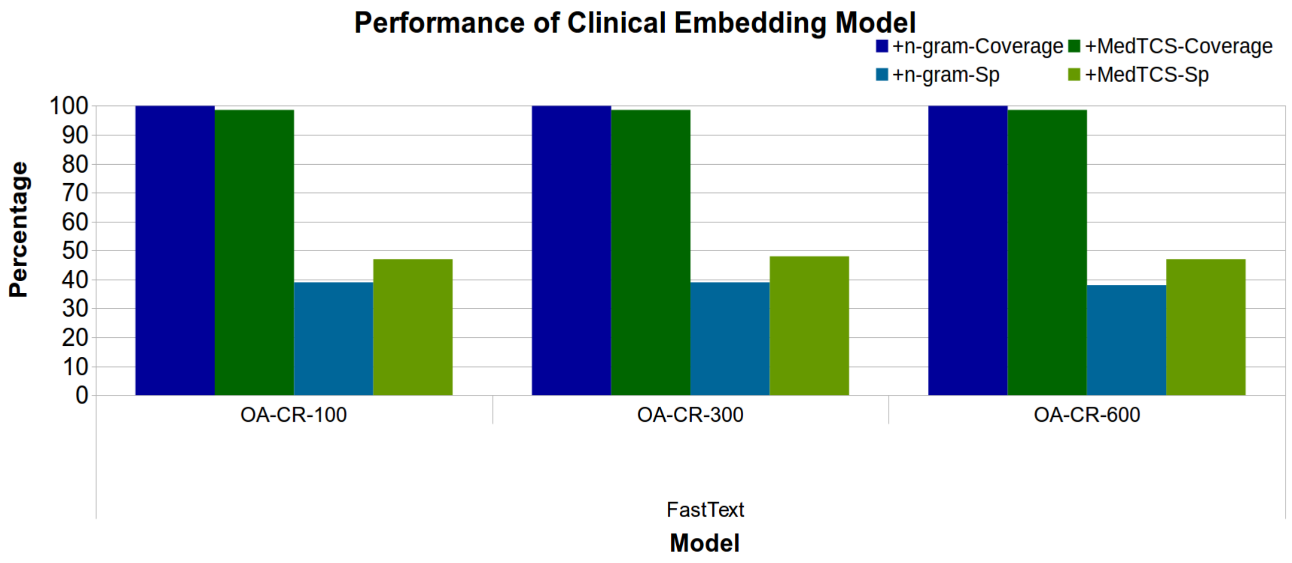

Figure 2. *Comparison of performance variations in clinical embedding model between n-gram and MedTCS module for OOV problem in UMNSRS-Similarity dataset.*

OOV words more reliably. Spearman-correlation scores of the MedTCS were improved from 0.39 $\rightarrow$ 0.47 as compared to the n-gram approach Figure 2.

The performance variations between the n-gram and MedTCS approaches to handle the out-of-vocabulary (OOV) problem on different variants of FastText clinical embedding models of OA-CR and OA-ALL Flamholz et al. (2022).

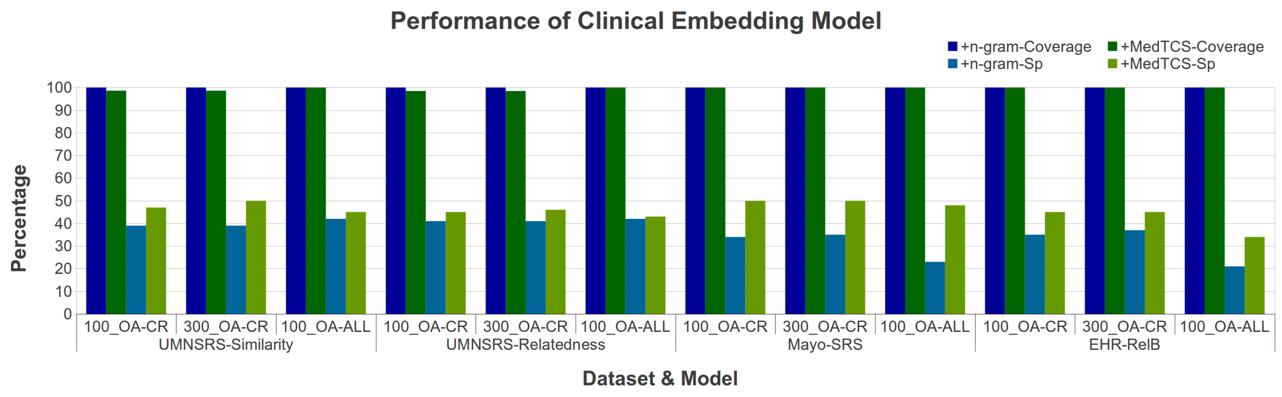

Figure 3. *Comparison of performance variations in clinical embedding model after adding MedTCS module for intrinsic evaluation.*

Thus, the MedTCS module enabled the OA-CR models Flamholz et al. (2022) to generate a vector for OOV terms from its search-space effectively.

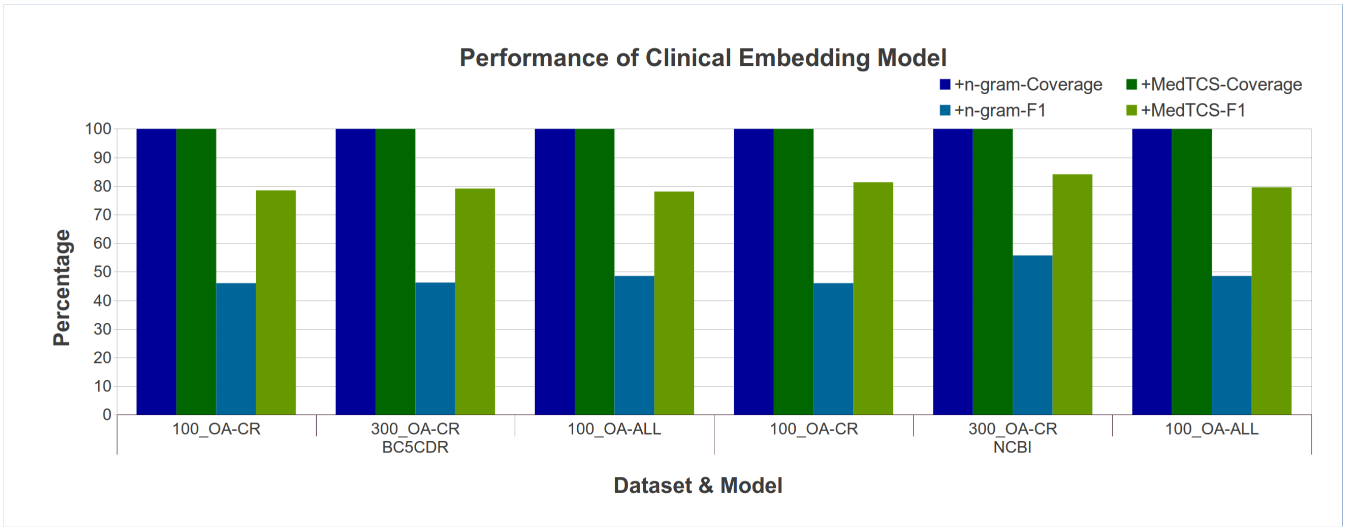

Figure 4. *Comparison of performance variations in clinical embedding model after adding MedTCS module for extrinsic evaluation.*

REFERENCES

Flamholz, Z. N., Crane-Droesch, A., Ungar, L. H., and Weissman, G. E. (2022). Word embeddings trained on published case reports are lightweight, effective for clinical tasks, and free of protected health information. *Journal of biomedical informatics* 125, 103971

Pakhomov, S., McInnes, B., Adam, T., Liu, Y., Pedersen, T., and Melton, G. B. (2010). Semantic similarity and relatedness between clinical terms: an experimental study. In *AMIA annual symposium proceedings* (American Medical Informatics Association), vol. 2010, 572
